# Perceptual Cue Weighting Is Influenced by the Listener's Gender and Subjective Evaluations of the Speaker: The Case of English Stop Voicing

**DOI:** 10.3389/fpsyg.2022.840291

**Published:** 2022-04-20

**Authors:** Alan C. L. Yu

**Affiliations:** Chicago Phonology Laboratory, Department of Linguistics, University of Chicago, Chicago, IL, United States

**Keywords:** speech perception, sociophonetics, cue weighting, English stop voicing, paralinguistic information, gender, personality traits, subjective evaluations

## Abstract

Speech categories are defined by multiple acoustic dimensions and their boundaries are generally fuzzy and ambiguous in part because listeners often give differential weighting to these cue dimensions during phonetic categorization. This study explored how a listener's perception of a speaker's socio-indexical and personality characteristics influences the listener's perceptual cue weighting. In a matched-guise study, three groups of listeners classified a series of gender-neutral /b/-/p/ continua that vary in VOT and F0 at the onset of the following vowel. Listeners were assigned to one of three prompt conditions (i.e., a visually male talker, a visually female talker, or audio-only) and rated the talker in terms of vocal (and facial, in the visual prompt conditions) gender prototypicality, attractiveness, friendliness, confidence, trustworthiness, and gayness. Male listeners and listeners who saw a male face showed less reliance on VOT compared to listeners in the other conditions. Listeners' visual evaluation of the talker also affected their weighting of VOT and onset F0 cues, although the effects of facial impressions differ depending on the gender of the listener. The results demonstrate that individual differences in perceptual cue weighting are modulated by the listener's gender and his/her subjective evaluation of the talker. These findings lend support for exemplar-based models of speech perception and production where socio-indexical features are encoded as a part of the episodic traces in the listeners' mental lexicon. This study also shed light on the relationship between individual variation in cue weighting and community-level sound change by demonstrating that VOT and onset F0 co-variation in North American English has acquired a certain degree of socio-indexical significance.

## 1. Introduction

Speech categories are defined by multiple acoustic dimensions. The acoustic and perceptual boundaries between speech categories are generally fuzzy in part because both speakers and listeners often give differential weighting to these dimensions in production and in perception. This study investigates how and why listeners may vary their perceptual weight of cues, with special focus on the voicing contrast in English initial stop, a prime example of the type of category fuzziness mentioned above.

The distinction between voiced and voiceless stops in English can be conveyed by as many as sixteen cues (Lisker, 1986). Voice onset time (VOT) and fundamental frequency (F0) at the onset of the following vowel, for example, have often been observed to co-vary in English word-initial plosives, with phonologically voiceless plosives followed by raised F0 at the vocalic onset, while phonologically voiced plosives (which are canonically realized with zero to weakly positive VOT) followed by lowered onset F0. Listeners have been found to be very sensitive to this type of onset F0 perturbations. Many studies have demonstrated that listeners can adjust their categorization of synthetic or digitally manipulated natural speech varying perceptually from voiced to voiceless stops depending on the F0 of the following vowel. Stimuli with lower F0's are more likely to be categorized as voiced whereas stimuli with higher F0's (but with otherwise identical acoustic characteristics) tend to be labeled as voiceless. A particular intriguing aspect of perceptual cue weighting, including the relative perceptual importance of VOT and onset F0 cues for stop voicing perception, is that it is not only language-specific (Schertz et al., 2015), there is also great individual-specific variation (Shultz et al., 2012; Clayards, 2018a) and such variation has been shown to be systematic across individuals (Idemaru et al., 2012; Schertz et al., 2015; Ou and Yu, 2021; Ou et al., 2021). What factors govern the differences in cue weighting between individuals remain under-investigated. In light of recent work that suggests socio-indexical information can influence speech perception, this study aims to elucidate the effects of listener's subjective evaluation of the talker on perceptual cue weighting, in particular the weighting between VOT and F0 for the English stop voicing contrast. The next section reviews important background information that motivates the current study. Section 3 introduces the experimental setup, followed by a discussion of the results in Section 4. Section 5 summarizes the study, discussing the implication of the present study for cue weighting research and for sound change theories.

## 2. Background

### 2.1. Sources of Individual Variability in Cue Weighting

Researchers have attempted to explain onset F0 perturbations as a reflex of aerodynamic (Ladefoged, 1967) and/or articulatory (Halle and Stevens, 1971; Ohala, 1973; Löfqvist et al., 1989) byproducts of stop voicing production. More recently, many have argued that onset F0 perturbations in English is actively controlled by speakers, perhaps to enhance this specific phonological contrast (Kingston and Diehl, 1994; Keyser and Stevens, 2006; Kingston, 2007; Solé, 2007; Hanson, 2009). Specifically, studies of onset F0 perturbations have found that the extent of onset F0 perturbations is not only language-specific (Hombert et al., 1979; Francis et al., 2006; Dmitrieva et al., 2015), but it can also vary quite extensively across individuals (Shultz et al., 2012; Chodroff and Wilson, 2018; Clayards, 2018b). Also consistent with the controlled phonetic interpretation of onset F0 perturbations is the context-dependency of onset F0 perturbations. Hanson (2009) observed that, in high pitch environment within a given speaker's F0 range, F0 is greatly increased following voiceless obstruents relative to a baseline F0, but not following voiced ones. In low-pitch environment, F0 is slightly increased relative to a baseline following all obstruents. She interpreted this difference in onset F0 perturbations in high vs. low pitch contexts as an indication of contrast enhancement since VOT is less distinctive in high pitch context than in low ones (see also Kirby et al., 2020). Echoing the variability observed in the production domain, the perceptual importance of these cues has also been found to be quite variable. Not only do listeners adjust their cue reliance in different contexts (Haggard et al., 1981; Repp, 1982) and when they are under different cognitive loads (Gordon et al., 1993), many studies have also found a trading relationship between the perceptual weightings of the VOT and F0 cues across listeners. Specifically, English listeners who rely on the VOT cue are found to rely less on the onset F0 cue, indicating a trading relation between these cues (Kapnoula, 2016; Kapnoula et al., 2017; Ou et al., 2021). Crucially, individual differences in cue weight have been shown to be stable across time (Idemaru et al., 2012; Schertz et al., 2015; Kapnoula, 2016) and across contrasts (Clayards, 2018a; Ou et al., 2021).

What factors govern the differences in cue weighting between individuals remains a largely unanswered question. Variability might stem from differences in individual perceptual experiences, as evidenced by perceptual learning experimental results showing that listeners can adjust their perceptual cue weights in accordance with the cue distributions in the exposure stimuli (e.g., Francis et al., 2008; Lehet and Holt, 2017; Zhang and Holt, 2018). An experience-driven approach to individual variation in cue weighting seems insufficient, however, given the often elusive mapping between perception and production of cue weights. While phonetic imitation studies have found that some speakers may adjust their VOT production when exposed to a model talker with a different VOT distribution, results from studies that look at direct correspondences between perceptual and production cue weighting have been mixed. Shultz et al. (2012), for example, investigated the use of VOT and F0 in producing and perceiving the English stop voicing contrast. While they found a significant negative correlation between VOT and onset F0 in production (see also Dmitrieva et al., 2015; but see Chodroff and Wilson, 2018; Clayards, 2018b, who did not find such a significant correlation in production), but did not find a significant correlation in the corresponding perceptual weights. They also did not find a significant correlation between perceptual and production cue weights. Schertz et al. (2015) examined native Korean speakers' perception and production of stop contrasts in their native language (L1) and second language (L2, English) and found that Korean listeners use different cue weighting strategies for both Korean and English stop voicing contrasts. They identified three general patterns among the L1 Korean listeners. The so-called “VOT group” classified stimuli with a long VOT as voiceless and a short VOT as voiced irrespective of F0, while the “F0 group” classified stimuli with high F0 as voiceless and low F0 as voiced irrespective of VOT. Finally, the “VOT+F0” group classified only stimuli with high F0 and long VOT as voiceless and all other stimuli as voiced. Of particular interest is that differences in perception were not predicted by individual variation in production patterns (Schertz et al., 2015). Such findings are problematic for input-driven accounts of speech categorization and cue weight setting that assume a tight perception-production loop since such models assume that speech classification and cue distributions are either estimated directly from the input (Pierrehumbert, 2002; Kronrod et al., 2016) or as a function of both the statistics of the input and the history of the learning system (Toscano and McMurray, 2010). Findings like those reported in Schertz et al. (2015) suggest that there might be other factors that mediate listeners' perceptual experiences that render the mapping between perception and production imperfect.

The fact that individual variation in cue weights is systematic across individuals (Idemaru et al., 2012; Schertz et al., 2015; Kapnoula, 2016; Ou et al., 2021) and not contrast-specific (Clayards, 2018a; Ou et al., 2021) suggests that such individual variability might stem from the influence of some general cognitive mechanism that modulates cue weights. Kong and Edwards (2016), for example, tied individual variability in perceptual cue trading between VOT and F0 to categorization gradience. Specifically, they found that listeners who exhibited a more gradient response pattern in a visual analog task also showed more sensitivity to F0 in an anticipatory eye movement task. Individual variability in categorization gradience might in turn stem from individual differences in neural encoding of the speech signal at the subcortical and cortical levels (Ou and Yu, 2021). Individual differences in cue weighting might also stem from individual variation in speech processing strategies. In their investigation of secondary cue weighting in two sets of English contrasts (/b/ vs. /p/ and /i/ vs. /ɪ/) using an eye-tracking paradigm, Ou et al. (2021) found that individuals who integrate secondary cues more extensively during processing are more likely to utilize a buffer processing strategy, suggesting a delayed reaction to the early-arriving cue until all relevant cues are available may facilitate the integration of multiple cues in the signal.

Another important source of individual variability that has yet to be explored in cue weighting research is the influence of socio-indexical and paralinguistic information on speech perception. The idea that socio-indexical information influences speech perception is not new *per se*. Strand (2000), for example, found that words are processed more quickly when the pitch of the talker is typical of his/her gender. Hay et al. (2006) investigated a case of merger in progress in New Zealand English (i.e., the merger of diphthongs /iɑ/ and /eɑ/) and found that the age and social class of the talker biased the listeners' perception of otherwise identical auditory stimuli. Staum Casasanto (2010) investigated the effect of listeners' experience with an ethnic dialect has on t/d deletion and found that listeners use social information about speakers (i.e., whether the face of the talker is Black or White) to make inferences about speech. Phonetic imitation/convergence research has also pointed to a significant influence of socio-indexical information on speech perception since whatever production adjustments in the direction of the model talker or interlocutors must presumably be perceptually detected in the first place. For example, Babel (2012) investigated the imitation of vowels in a lexical shadowing task and found that the degree to which vowels were imitated was subtly affected by how attractive the talker was rated by the participants; the listeners were given either no image, or saw either a Black talker or a White talker. Yu et al. (2013) investigated the imitation of VOT and found that the extent of phonetic imitation is modulated by the participant's subjective attitude toward the model talker, the participant's personality trait of openness, and the autistic-like trait associated with attention switching.

Evidence of socio-indexical information influencing speech perception and phonetic imitation/convergence lends support for models of speech perception and production where socio-indexical features are encoded as a part of the episodic traces in the listeners' mental lexicon and the activation of socio-indexical information will result in the activation of episodic traces that are consistent with, or linked to, the social category or feature (e.g., Sumner et al., 2014; Babel and Russell, 2015; McGowan, 2015). Thus, when a talker is perceived to be of a particular gender or has certain personality features such as being attractive or friendly, the listener's perception will be primed to interpret the speech signal in ways that are consistent with the social expectation (see also similar accounts under the rational exemplar-based model or the ideal adapter framework Kleinschmidt and Jaeger, 2015; Myslin and Levy, 2016; Kleinschmidt et al., 2018).

### 2.2. The Socio-Indexical and Paralinguistic Characteristics of VOT and F0

In addition to the fact that the likelihood of VOT imitation can be modulated by a listener's subjective evaluation of the talker, various converging evidence further lends support to the idea that the socio-indexical and paralinguistic characteristics of the talker may influence listeners' perception of English stop voicing. To begin with, Swartz (1992) reported that females have longer VOTs than males (see also Ryalls et al., 1997; Whiteside and Irving, 1997, 1998; Koenig, 2000; Whiteside and Marshall, 2001; Whiteside et al., 2004b; Robb et al., 2005; cf. Morris et al., 2008). Some attributed this gender-based VOT difference to anatomical differences in phonatory apparatus between genders, such as men's wider supraglottic space and women's shorter and stiffer vocal folds (e.g., Swartz, 1992; Whiteside and Irving, 1997, 1998; Koenig, 2000; Oh, 2011), others hypothesized that the pattern might stem from voicing contrast optimization in female speech (Whiteside and Irving, 1998). The physiological explanation is undermined by the fact that the same gender difference is not uniformly observed cross-linguistically (Oh, 2011; Lundeborg et al., 2012; Li, 2013; Reddy et al., 2013; Peng et al., 2014), further pointing to the potential socio-indexical relevance of this gender-based VOT difference in English. VOT is also reported to vary according to women's menstrual cycle; women who are at their reproductive peaks have longer VOTs than those at their lowest fertility levels (Whiteside et al., 2004a; Wadnerkar et al., 2006). Since women at the reproductive peaks of their menstral cycle are rated as more vocally attractive, Babel et al. (2014) reasoned that the increase in VOT, which could increase clarity in stop voicing contrasts, might also influence attractiveness judgments. It should be noted that, in clear speech, a mode of speaking that is associated with increased articulatory efforts, VOT for voiceless stops in English has also been found to be lengthened while the VOT for voiced stops remain unchanged (Smiljanić and Bradlow, 2008).

F0 also carries a wealth of social information about a person. To begin with, pitch, one of the most perceptually salient feature of human voice (Banse and Scherer, 1996), is about half as high in men as it is in women (Titze, 2000). The pitch of voice is inversely correlated with perceived dominance; the lower the voice pitch, the greater the perceived dominance (Puts et al., 2006). Adjusted for the effects of sex and age, Stern et al. (2021) found that participants with lower voice pitch self-report as lower on neuroticism, but higher on dominance, extraversion, and openness to experience, as well as more unrestricted on sociosexual orientation, sociosexual behavior, sociosexual attitudes, and sociosexual desire. Paralinguistic intonational meanings have been argued to be grounded in terms of the Frequency Code (Ohala, 1983, 1984; Chen et al., 2004a), which exploits the link between larynx size and vibration rates of the vocal cords for the expression of power relations, and the Effort Code (Gussenhoven, 2002), which refers to the positive correlation between articulatory efforts and articulatory precision (de Jong, 1995). Specifically, higher pitch has more affective interpretations, which include “uncertain”, “feminine”, “submission”, “friendly”, “polite”, and “vulnerable”, while lower pitch has “certain”, “masculine”, “dominant”, “confident”, “protective”, and “aggressive” interpretations (Gussenhoven, 2002; Chen et al., 2004a,b). Greater pitch excursion is also associated with informational interpretations such as “emphatic” and “significant” and affective interpretation of “surprised” and “agitated” and even “obliging” (Gussenhoven, 2002).

Perceived sexual orientation has also been associated with variation in VOT and F0. More-gay sounding men, for example, has been found to produce stop consonants with longer voice-onset times than less-gay sounding men (Smyth and Rogers, 2002). Gayness ratings were strongly correlated with independently made judgments of perceived intonational variability, even though mean F0 and F0 variability did not predict gayness ratings (Smyth et al., 2003). In particular, the voices that were rated as gay-sounding by one group of listeners were rated by an independent group of listeners as having greater F0 modulation; conversely, listeners were more likely to falsely judge a voice as having greater F0 modulation if that voice had been judged by an independent group to be gay-sounding.

As noted above, the difference in onset F0 after voiced and voiceless stops (onset F0 perturbations), is found to be greater in higher global F0 contexts than in lower ones (Hanson, 2009; Kirby et al., 2020), we hypothesize that listeners might make use of such an association when processing onset F0 perturbations produced by talkers of different genders or talkers associated with certain paralinguistic features given their different F0 profiles. There is some suggestive evidence to support this hypothesis. Zhang and Holt (2018), for example, found that global F0 differences can influence stop voicing categorization, but this F0 effect is more apparent when the talker is perceived to be female. Specifically, in a series of perceptual learning experiments, they recruited two groups of listeners, half presented with high vs. mid F0 global contours (the high F0 range group), while the other half with the mid and low F0 contours (the low F0 range group). They found significant differences in voicing responses depending on the global F0 height, with higher F0 contours associated with more voiceless response than lower F0 contours. Crucially, in two followup studies, they manipulated the perceived gender of the talker(s) acoustically (via changes in the formants of the stimuli) and visually. For the “female” voice stimuli (i.e., high F0 range stimuli with female-like formant values), listeners showed a difference in /p/ response according to the high or low global F0 profile of the stimuli within the “female” global pitch range, but no comparable global F0-dependent /p/ response difference was observed with the “male” stimuli (i.e., low F0 range stimuli with male-like formant values). These findings suggest that the perceived gender of the talker influences the effects of global F0 have on English stop voicing perception.

To be sure, Zhang and Holt's study did not address onset F0 perturbations specifically as the F0 differences are not localized to the onset of the vowel. Thus, it remains unclear if the gender of the talker would influence the effect of onset F0 perturbation on stop voicing perception. Also, since the participants' gender evaluation of the talkers was not examined, it is difficult to ascertain whether the participants' perception of the talker gender matched the expectation of the experimenter. Finally, their perceived gender findings were based on a within-subject design where listeners were presented with both “male” (i.e., low F0 range) and “female” (i.e., high F0 range) stimuli within the same block. This design raises the possibility that the different rates of /p/ responses across the perceived gender conditions might come about as a result of a contrast effect. That is, listeners only adjusted their expectation when they encountered both high and low F0 talkers, but not when they listened to a single talker with small variation in global F0.

The present study built on these earlier findings and examined whether the perceived gender and the listener's impression of the talker's facial and vocal features influence listeners' perception of word-initial voiced and voiceless stops in English using a matched-guise design (Lambert et al., 1960; Zahn and Hopper, 1985). In particular, three groups of listeners classified the same set of acoustic stimuli. Two groups were given a visual prompt of the talker: one group of participants in the visual prompt condition was presented with an image of a prototypical male and the other group with the image of a prototypical female. Given that previous studies have shown that rapid evaluative inferences based solely on facial and vocal information can exert a significant influence on the perceiver/listener behavior [e.g., sales (Jacob et al., 2011), stock market returns (Mayew and Venkatachalam, 2012), wage penalty (Grogger, 2011; Rickford et al., 2015), election outcomes (Todorov et al., 2005; Klofstad, 2017), housing market interactions (Purnell et al., 1999), likelihood of vowel imitation (Babel, 2012), and language processing speed (Staum Casasanto, 2010)], we hypothesize that listeners would adjust their perceptual cue weights if they are aware of the association between the VOT/onset F0 covariation on the one hand and the socio-indexical and personality characteristics on the other. We also aimed to examine whether facial and vocal impressions exert similar influences on the listener's cue weighting. Previous literature reported conflicting findings concerning the strength of facial and vocal impressions. While some studies reported stronger effects of facial impression over vocal impressions (e.g., Klofstad, 2017; Hou and Ye, 2019), others found the opposite tendency (Schroeder and Epley, 2015).

## 3. Methods and Materials

### 3.1. Participants

304 native speakers of American English were recruited to participate in this study on Prolific (https://www.prolific.co/), a crowd-sourcing platform for online studies that, in addition to confirming the identity of each participant, gathers extensive self-reported demographic information from each participant for prescreening purposes. Participation in this study was limited to individuals who reported being 18–40 years old, native speakers of English, residents of the United States, right-hand dominant, with no history of hearing, language, neurological, or mental disorders. In the end, a total of 237 participants' responses were analyzed. Sixty-seven were excluded from the study due to failure to pass the headphone screen (*N* = 23) or failure to meet compliance checks (i.e., not a native speaker of English, participated in more than one prompt condition, and/or have a history of one or more of the following: speech/hearing/language disorders, dyslexia, autism, substance dependence, stroke, mental retardation, traumatic brain injury with greater than 1 h loss of consciousness, multiple sclerosis, Parkinson's disease, Alzheimer's disease, Huntington's disease, schizophrenia, bipolar, ADHD, or current major depression; *N* = 44). This attrition rate is consistent with other web-based studies (Thomas and Clifford, 2017; Woods et al., 2017; Brown et al., 2018; Giovannone and Theodore, 2021).

The cohort is roughly gender-balanced in each prompt condition. Table 1 provides a detailed gender breakdown of the number of participants within each condition. The median age is 25 (Mean = 26.62, SD = 6.37). Additionally, 87 participants reported having some musical training and 128 reported speaking or having studied another language other than English. The participants were paid $2 for their participation in the study; the study lasted, on average, around 10 min.

**Table 1:** Mean ratings (and standard deviations in parentheses) for perceived vocal gender, attractiveness, friendliness, confidence, trustworthiness, and gayness in the two face conditions arranged by the gender of the participants.

**Condition**	**Listener**	***N***	**Gender**	**Attractive**	**Friendly**	**Confident**	**Trustworthy**	**Gay**
AudioOnly	Female	39	67 (16)	27 (17)	57 (22)	46 (20)	49 (17)	52 (23)
AudioOnly	Male	47	67 (20)	30 (22)	54 (23)	44 (23)	50 (22)	48 (27)
Female	Female	36	63 (15)	30 (18)	47 (21)	45 (18)	48 (19)	58 (22)
Female	Male	40	66 (18)	31 (20)	47 (18)	48 (19)	49 (19)	50 (24)
Male	Female	35	42 (16)	44 (20)	62 (16)	55 (16)	54 (14)	57 (11)
Male	Male	40	44 (22)	47 (20)	60 (17)	47 (18)	55 (15)	57 (22)

### 3.2. Stimuli

In order to create a gender-neutral voice suitable for the study, a gender prototypicality rating task was conducted. The stimuli, based on recordings of /b/ “bear” and /p/ “pear” produced by a male native speaker of American English, were generated by modifying the recordings in terms of Formant Shift and Pitch Shift, using a custom-written script from Xu et al. (2013) that applied the “Change Gender” function in the Praat program (https://doi.org/10.1371/journal.pone.0062397.s002). In total, 25 stimuli were prepared, that is, 5 formant shift ratios (0.8, 0.9, 1, 1.1, 1.2) × 5 pitch shifts (−5, −4, 0, 4, 5). The “Change Gender” function in Praat shifts formant frequencies as a ratio of the original sound via manipulation of sampling frequency. The manipulation shifted the formant frequencies in the original speech token toward a more exaggerated female voice (formant shift ratios of 1.1 and 1.2) or toward a more male voice (formant shift ratios of 0.8 and 0.9). Prior to creating the different voices, the F0 of the original speech token was first resynthesized to have a flat F0 contour at 154 Hz. Ten participants, recruited on Prolific, listened to all 25 speech tokens in a randomized order to decide how male- or female-sounding a voice is by adjusting a sliding scale that ranges from prototypical female to prototypical male. The polarity of the scale was counter-balanced across participants. The voice with formant shift ratio of 1.1 and F0 at 154 Hz was chosen as the stimuli for the main experiment because it was rated most neutral (i.e., the midpoint of the gender prototypicality scale) most often and most consistently (mean = 49.2, sd = 5.5).

A 7-step /b/ to /p/ VOT continuum was created out of the selected gender-neutral voice “bear”/“pear” tokens by cross-splicing aspiration from the naturally produced voiceless bilabial /p/ in “pear” to the voiced bilabial /b/ in “bear” at 7 ms increments using the custom script described in Winn (2020). Each step on the continuum was given one of two F0 contours where F0 began at either 134 or 174 Hz and fell (or rose) linearly until 154 Hz at the 75 ms from vowel onset. The 7 (VOT) × 2 (F0 target) design yielded 14 distinct items. The intensity of all stimuli was normalized to the same level.

### 3.3. Procedure

Both the gender prototypicality rating task and the main experiment were hosted on Qualtrics. To ensure that participants were wearing headphones, all participants first passed a headphone screen developed by Woods et al. (2017). In this task, listeners judge which sound in a series of three pure tones is the quietest, with one sound presented out of phase on the stereo channels. This task is designed to be easy when the participant is wearing headphones or earbuds, but extremely challenging over loud speakers due to phase-cancellation. If participants did not correctly pass 5 out of 6 trials, they were reminded to wear headphones and asked to repeat the task. If they failed the headphone check twice, they were asked to return the task in order to receive partial compensation for their efforts.

After the headphone check, participants completed a short demographic survey to gather any information not made available through Prolific. This is followed by either one or two first impression rating task(s) depending on the prompt condition. Participants were randomly assigned to either a condition with visual prompt or one without. Those in the visual prompt conditions were shown either a prototypical male or prototypical female face selected from the Chicago Face Database (Ma et al., 2015). The specific faces can be found in the Supplementary Data. Participants in the visual prompt conditions completed two first impression rating tasks. The first rating task asked the participant to rate the talker faces in terms of their gender-prototypicality, the attractiveness, friendliness, confidence, trustworthiness and whether the individual looked gay. These personality attributes were selected in part based on previous research on listener's perceptual evaluations of linguistic variables (Eckert, 2008; Campbell-Kibler, 2009, 2010; McAleer et al., 2014) as well as their the associations between the specific attributes and the two phonetic dimensions targeted in this study as reviewed in the Introduction.

The participants then listened to the voice of the talker and rated the voice on the same attributes as the visual impression survey. The stimulus heard was a recording of the word “bear” with zero VOT (i.e., step 1 of the VOT continuum) with a rising F0 onset. Participants in the “audio-only” condition completed only the vocal impression rating task.

Following the rating task(s), the participants were asked to listen to the target stimuli and determine whether they heard the word “bear” or “pear” by clicking on the corresponding picture. Each participant classified 112 stimuli (7 VOT steps × 2 F0 targets × 8 blocks). The trials were split into eight blocks, each consisted of the fourteen target stimuli randomly ordered within each block. The instructions (and the talker image in the visual prompt conditions) were repeated at the beginning of each block. The positions of the response pictures were counterbalanced across blocks. To encourage the participant to stay alert, the participant completed a ten-question Short Autism Spectrum Quotient (Allison et al., 2012) after four blocks of the categorization task. Following the completion of all eight blocks of the categorization task, participants completed the headphone screen again before exiting the task.

### 3.4. Predictions

Before diving into the results, it is worth laying out some *a priori* predictions based on the literature reviewed above. Concerning gender-based differences, we advance three potential hypotheses. As alluded to in Section 2, from the perspective of episodic/exemplar-based models of speech perception and production, when a talker is perceived to be of a particular gender or has certain paralinguistic features such as being attractive or friendly, the activation of the relevant socio-indexical/paralinguistic information will result in the activation of episodic traces that are consistent with, or linked to, the social category or paralinguistic feature (e.g., Sumner et al., 2014; Babel and Russell, 2015; McGowan, 2015). This means that the listener's perception will be primed to interpret the speech signal in ways that are consistent with the social expectation. Specifically, given that VOT is less distinct between voiced and voiceless stops in word-initial position in males compared to females, we expect listeners to be sensitive to this gender difference in VOT realization and exhibit less reliance on the VOT cue when listening to a talker who is perceived to be male than when the talker is perceived to be female. Assuming that there is a perception-production loop, where stored perceptual experiences are weighted by social and attentional factors and such perceptual exemplars are drawn upon to generate production targets (Pierrehumbert, 2002), we expect that male listeners may also rely less on the VOT cue than female listeners, if male listeners mirror the production tendencies of male speakers. Furthermore, to the extent that the perceptual cue weights for VOT and F0 are in a trading relation, we expect listeners who assign less weight to the VOT would rely more on F0 in stop voicing classification.

Turning to potential effects of socio-indexical and paralinguistic information on the relative cue weighting between VOT and F0, recall that, within a given talker's F0 range, onset F0 perturbations are larger when the global F0 environment is high and VOT for voiceless stops are shorter. To the extent that femininity, friendliness, trustworthiness are associated with higher overall F0 and more dynamic F0 excursion, we hypothesize that listeners may rely more on F0 information and less on VOT information for stop voicing perception when the talker is thought to be associated with those personality characteristics. To the extent that attractive, confident, or gay-sounding voices are associated with greater VOT differences between voiced and voiceless stops, we expect listeners to rely more on the VOT cue when listening to talkers who are rated as more attractive and confident.

## 4. Results

We begin the presentation of the results of the study by first examining the effects of vocal impressions on the identification of stop voicing in English in Section 4.1 since visual information is only relevant in two of the three prompt conditions. Section 4.2 presents findings from the visual prompt conditions.

### 4.1. Results From All Prompt Conditions

**Figure 1:**
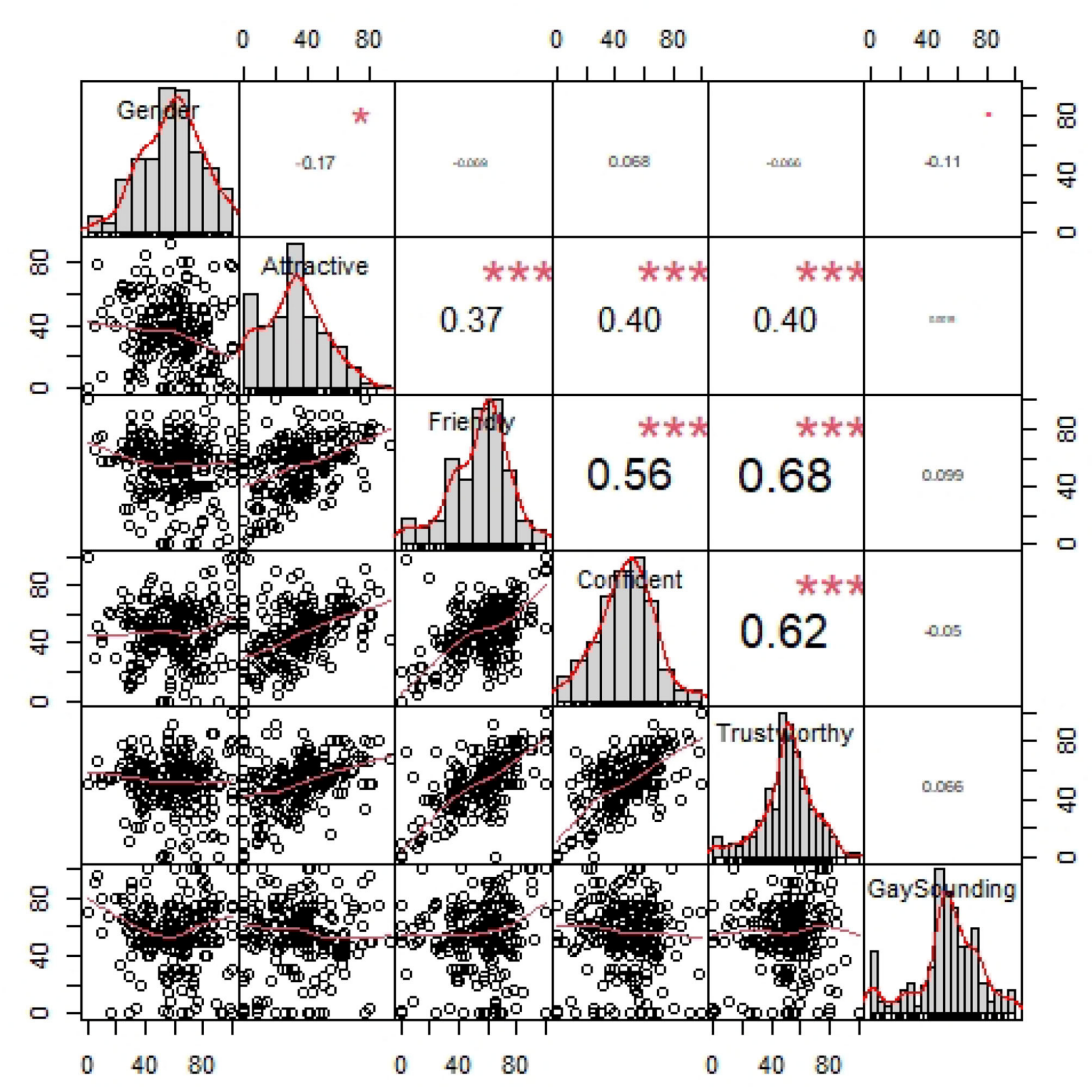
Correlations between the ratings across different vocal attributes. Each point corresponds to the ratings of a participant. ^***^p < 0.001; ^*^p < 0.05.

Before introducing the first regression model, Table 1 summarizes the vocal impression ratings. Several aspects of the rating data are noteworthy. Not only is there great variability in how the participants rated the talker's vocal gender prototypicality, as illustrated in Figure 1, there is also a great deal of variation in ratings for each dimension, as well as variation in how the attributes relate to each other. Specifically, there are strong positive correlations between Attractiveness, Friendliness, Confidence, and Trustworthy and a negative correlation between Gender and Friendliness, as seen Figure 1.

#### 4.1.1. Principal Component Analysis of the Vocal Impression Ratings

Given the highly correlated nature of some of the vocal impression attributes, in an effort to reduce the dimensionality of the mapping between vocal impressions and perceptual responses, rather than analyzing the vocal impression ratings individually, an integrated cue-combination approach was taken such that the vocal impression ratings were first submitted to a principal component analysis (PCA) to obtain linear combinations of these vocal impression ratings that would capture the maximum variation. The specifics of the PCA are as follows: the vocal impression ratings, which were z-scored, were analyzed using the prcomp() function in R, which performs a principal component analysis on a given data matrix; principal components with an eigenvalue greater than 1 were selected for the regression analysis (Kaiser, 1961). The relative weighting and proportion of variance for each component for the vocal attributes are summarized in Table 2. The optimal linear combination (PC1), which accounts for 42% of the variance, and the 2nd component (PC2), which accounts for 19% of the variance, were selected as independent variables for the analysis below; the first two components collectively account for around 62% of the variance. PC1 has strong loadings for vocal attractiveness, friendliness, confidence, and trustworthiness, which can be characterized as “vocal appeal”. PC2, on the other hand, is dominated by voice gender, confidence, and gay-sounding, which might be characterized as gender stereotypicality.

**Table 2:** The cumulative proportion of variance accounted for and loadings from the PCA of the vocal impression ratings.

	**PC1**	**PC2**	**PC3**	**PC4**	**PC5**	**PC6**
Vocal gender	−0.07	0.73	0.55	0.36	0.18	−0.05
Vocal attractiveness	0.40	−0.13	−0.36	0.82	0.14	−0.00
Vocal friendliness	0.53	−0.02	0.15	−0.29	0.55	0.56
Vocal confidence	0.51	0.25	0.05	−0.05	−0.78	0.26
Vocal trustworthiness	0.54	0.03	0.09	−0.25	0.12	−0.79
Vocal gay-sounding	0.05	−0.63	0.73	0.23	−0.14	0.01
Standard deviation	1.60	1.07	0.97	0.80	0.64	0.56
Proportion of variation	0.42	0.19	0.16	0.11	0.07	0.05
Cumulative proportion	0.42	0.62	0.77	0.88	0.95	1.00

#### 4.1.2. Model 1

Listeners' responses (/b/ = 0, /p/ = 1) were modeled with logistic mixed effects regressions using the glmer() function in the lme4 package (Bates et al., 2015) in R. The fixed effect predictors included in the model were trial block (Block: 1–8), VOT continuum step (VOT: 1-7), onset F0 (F0: High or Low), prompt Conditions (Helmert-coded: contrast 1 = audio only vs. visual prompt; contrast 2 = Male Face vs. Female Face), and the two PCs of the vocal impression ratings. The model also included the participant's Gender (Male vs. Female) as a between-subject factor given that effects of facial and vocal impressions on listener behavior have been found to be gender-differentiated (Babel, 2012; Chen et al., 2016). All continuous variables (i.e., Block, VOT, PC1, and PC2) were z-scored. Unless otherwise specified, categorical variables were sum-coded. The model also included all possible interactions between the fixed effects predictors other than Block as well as by-subject random intercepts and by-subject random slopes for Block, VOT, and F0, as well as the interaction between VOT and F0.

Model selection started with the maximal model with all possible interactions between fixed factors (the PCs of the vocal attributes did not interact with each other, however) as well as the random intercepts and slopes, and proceeded by comparing between models with and without the inclusion of a fixed/random factor and/or interaction. Predictors that do not improve model-likelihood significantly were dropped. In the end, neither PC1 nor PC2 of the vocal attributes was retained following this model selection procedure. The complete model in lme4 format is: Response (pear = 1) ~ Block + VOT * F0 + VOT * Gender + VOT * Condition + (1 + Block + VOT * F0|Participant).

**Figure 2:**
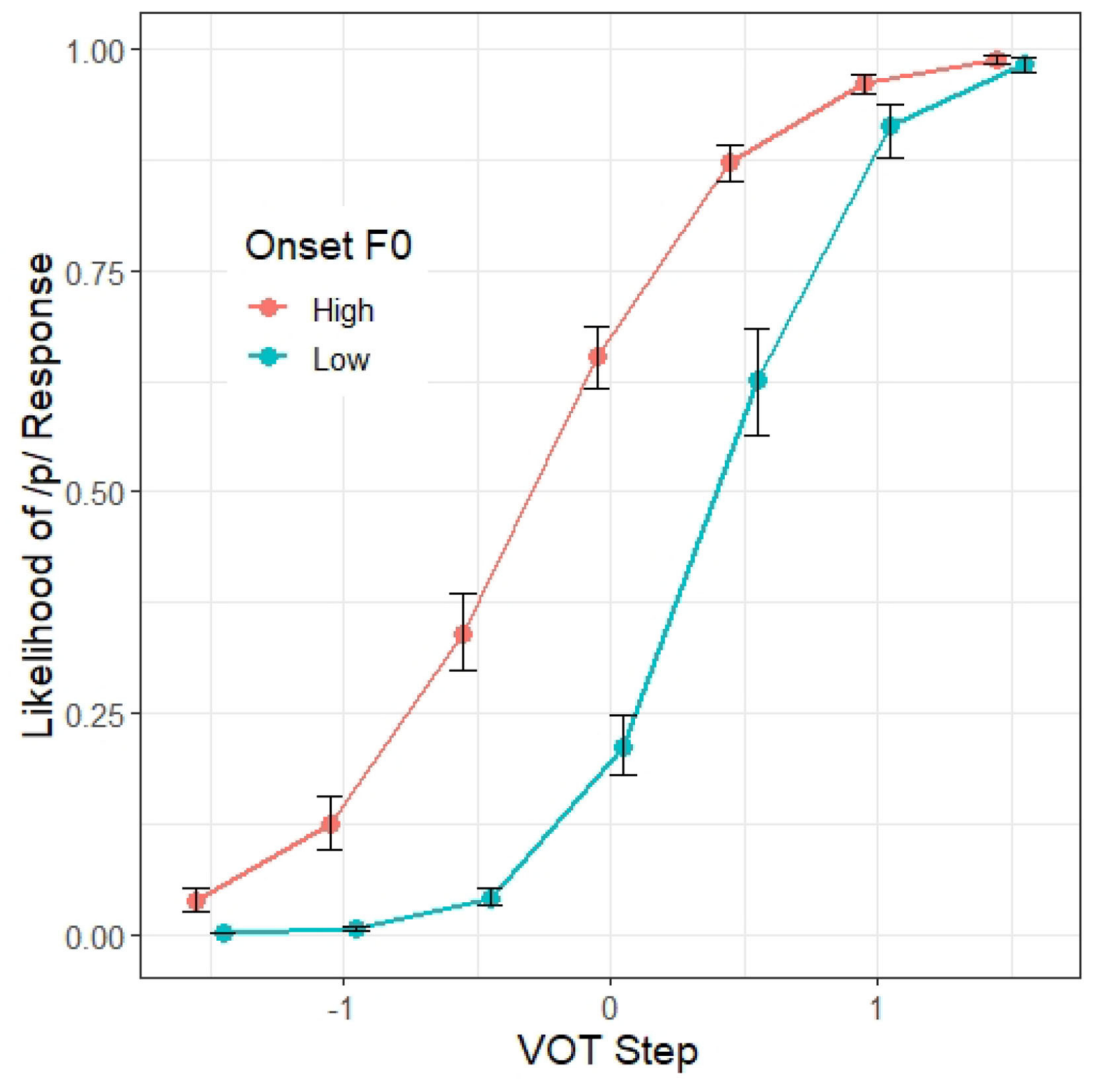
Model 1 predictions of the probability of a /p/ response (y axis) at different steps on the VOT continuum (x axis) and F0 targets. The error bars indicate 95% confidence intervals.

A summary of the first regression model, Model 1, appears in Table 3. As expected, VOT is a significant predictor (β = 3.13, *z* = 23.66, *p* < 0.001) as well as onset F0 (β = 0.97, *z* = 21.57, *p* < 0.001), suggesting that /p/ responses are more likely when VOT is longer and when the onset F0 is higher. There is also a significant interaction between VOT and onset F0 (β = −0.53, *z* = −14.26, *p* < 0.001), suggesting that the likelihood of a /p/ response along the VOT continuum varies depending on the onset F0. Visual inspection of Figure 2 shows that the F0 effect on /p/-response is strongest within the VOT range where VOT is not the most informative cue (i.e., the middle of the VOT continuum). There is also a significant effect of Block (β = 0.09, *z* = 3.21, *p* < 0.01), suggesting that the participants are more likely to respond /p/ as the experiment progressed.

**Table 3:** Estimates for all predictors in Model 1.

	**Model 1**
Intercept	−0.35(0.08)^***^
VOT	3.13(0.13)^***^
F0	0.97(0.05)^***^
Gender	−0.13(0.07)
Condition_*A*/*AV*_	−0.03(0.15)
Condition_*M*/*F*_	0.02(0.18)
Block	0.09(0.03)^**^
VOT:F0	−0.53(0.04)^***^
VOT:Gender	0.23(0.11)^*^
VOT:Condition_*A*/*AV*_	0.45(0.23)
VOT:Condition_*M*/*F*_	−0.67(0.28)^*^
AIC	18431.06
BIC	18643.91
Log Likelihood	−9189.53
Num. obs.	26544
Num. groups: Participant	237
Var: Participant Intercept	1.36
Var: Participant Block	0.08
Var: Participant F0	0.34
Var: Participant VOT	3.64
Var: Participant F0:VOT	0.11

*Gender refers to the gender of the participant. Condition_A/AV_, audio only vs. visual prompt; Condition_M/F_, Male Face vs. Female Face*.

*** *p < 0.001*;

** *p < 0.01*;

* *p < 0.05*.

**Figure 3:**
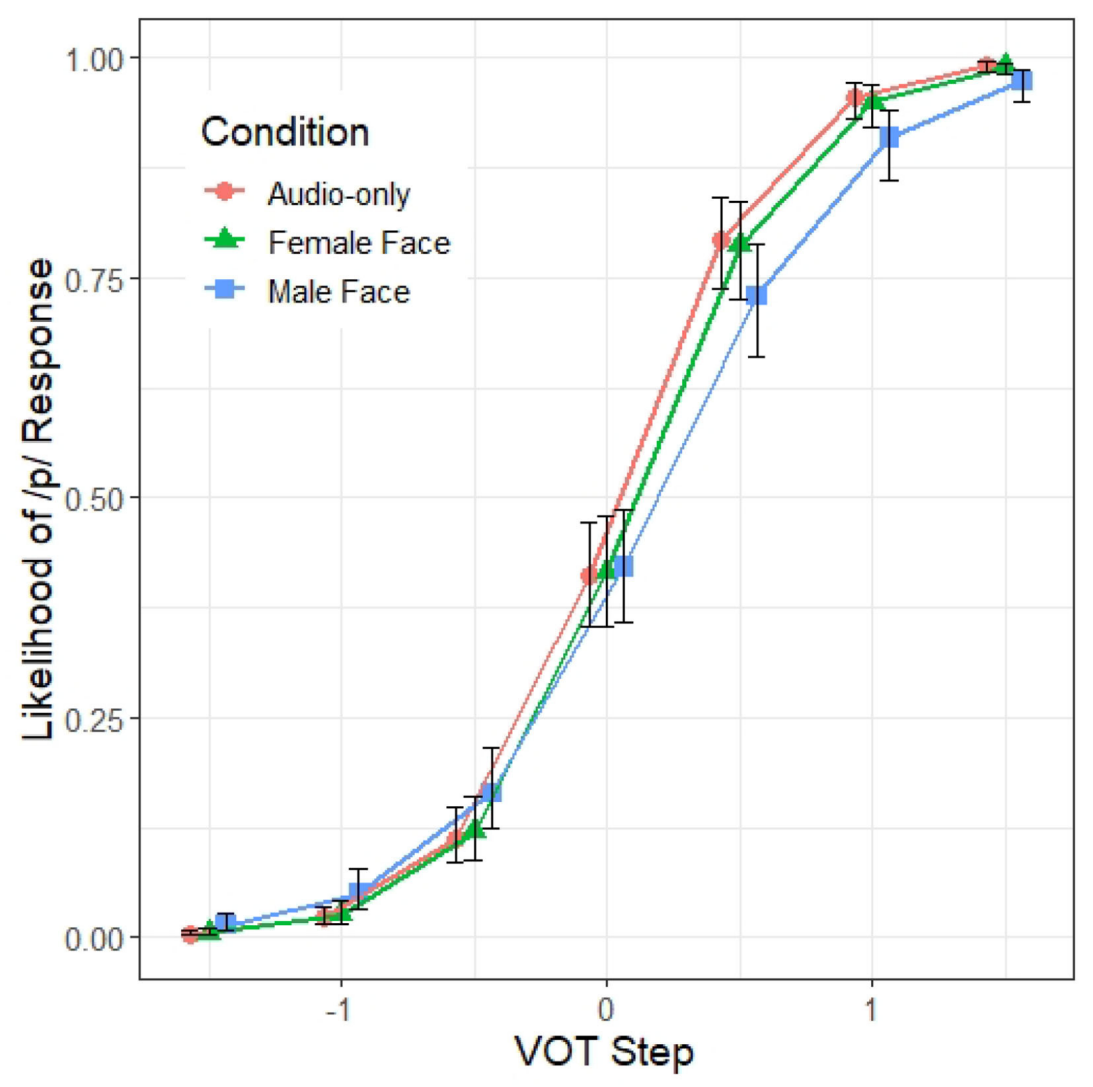
Model 1 predictions of the probability of a /p/ response (y axis) at different steps along the VOT continuum (x axis) and across the three prompt conditions. The error bars indicate 95% confidence intervals.

There is a significant interaction between VOT and Condition_*M*/*F*_ (β = −0.67, *z* = −2.43, *p* < 0.05). As illustrated in Figure 3, the classification function along the VOT dimension in the male face condition is shallower than in the female face condition. Specifically, the listeners in the male face condition are less likely to hear /p/ toward the /p/ end of the VOT continuum than those in the female face condition, suggesting that listeners in the male talker condition are less reliant on VOT as a cue for determining stop voicing. A separate model with the Condition treatment-coded with the audio-only condition as the baseline level showed that the response pattern from the audio-only condition differs significantly only from the male face condition, and not from the female face condition, suggesting that the VOT x Condition interaction is driven by the shallower VOT response pattern found in the male face condition.

**Figure 4:**
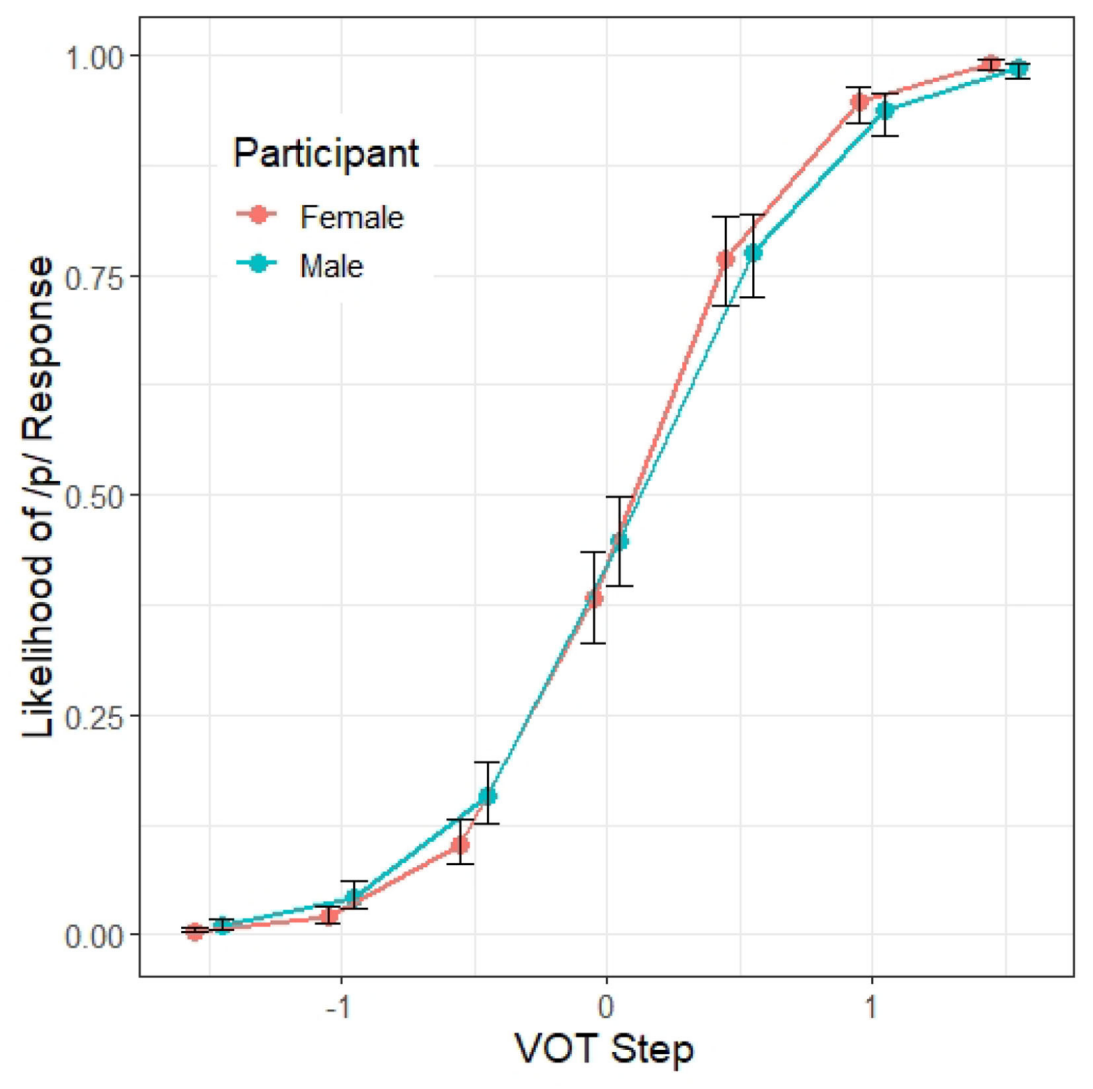
Model 1 predictions of the probability of a /p/ response (y axis) at different steps along the VOT continuum (x axis) by the gender of the participants. The error bars indicate 95% confidence intervals.

There is also a significant interaction between VOT and participant Gender (β = 0.23, *z* = 2.04, *p* < 0.05). Similar to the effect of Condition, as illustrated in Figure 4, male participants showed a shallower VOT slope than the female participants, suggesting that male listeners are less reliant on the VOT cue than the female listeners.

#### 4.1.3. Interim Summary

The fact that the stop voicing categorization along the VOT dimension is affected by the prompt manipulation and the gender of the listener suggests that the listeners are not evaluating the speech signal in a vacuum. In accordance with our hypothesis, listeners are less reliant on VOT (as indexed by the coefficient of the VOT factor in the model) in classifying the stop voicing when the participant saw a prototypical male talker face. Also consistent is the finding that male *listeners* are less likely to rely on VOT as a cue for stop voicing. As noted earlier, VOT tends to be shorter in male than in female (e.g., Swartz, 1992; Robb et al., 2005), which means that the contrast between voiced and voiceless stops in males is more endangered in general. From the perspective of exemplar-based models that allow socio-indexical information to be encoded with each perceptual exemplar (e.g., Babel and Russell, 2015; McGowan, 2015), when the listeners were prompted to think that they were listening to a male talker, they might be activating perceptual exemplars that are consistent with male talkers and adjusting their expectations, making allowance for more ambiguities in their VOT classification (hence the shallower slope) to reflect their past perceptual experiences. Male listeners also rely less on VOT presumably because they are more attuned to the skewed VOT distribution in men as a result of the perception-production loop.

Our hypothesis about potential cue trading between VOT and F0 did not find support from the Model 1 results. The fact that VOT is modulated by the visual prompt manipulation but not F0 is surprising as the downweighting of the VOT cue by the listeners in the male face condition is expected to show a corresponding upweighting of F0 in the same face condition if VOT and F0 were in a trading relationship. Also unexpected is the lack of a significant vocal impression effect on cue weighting. One possible explanation for these findings might pertain to the stronger influence of visual impression over vocal ones on speech perception. Note though that the visual prompt effect is mainly driven by the male face condition, so the visual prompt manipulation alone is not likely to be sufficient to explain the mute presence of vocal impression. To this end, it is worth noting that the gender rating of the talker in the “audio-only” condition skewed toward the masculine-end of the gender prototypicality continuum (i.e., the average gender prototypicality score is 67 on a scale where 0 indexes most female-like and 100 indexes most male-like), suggesting that the talker voice might not be as gender-neutral as we had assumed based on the results of the stimulus selection task; recall that stimulus selection task showed that the chosen voice has an average gender prototypicality score of 49.2 with a standard variation of 5.5. The mute presence of vocal impression effects might have been influenced by the perceived gender-biased nature of the voice, which could have reduced the variance needed to detect any vocal impression effects.

To be sure, there is a marked difference in gender prototypicality across the two visual prompt conditions. That is, the participants in the male face condition rated the talker as less masculine-sounding than in the female face condition (mean voice gender rating in the male face condition = 42.95 vs. female face = 64.55). These findings suggest that the visual prompts had an impact on how the listeners evaluated the voices; the voice was perceived to be more feminine when the participants were shown a male face and more masculine when the participants saw a female face. Listeners also did not process the visual information of the talker necessarily in the same way, particularly when it comes to perceived gender assumptions and visual first impression judgments. For example, there is quite a bit of variability in voice gender rating in both face conditions—male face: SD = 19.25, range = 0–100 vs. female face: SD = 16.27, range = 29–100. To examine in more depth the impact of the visual prompts on listeners' reliance on VOT and onset F0, the next section looks at whether and how the participants' visual impressions on the talker influence the participants' perceptual behavior.

### 4.2. Results From the Visual Prompt Conditions: Model 2

The last section demonstrated that the participants' reliance on VOT is impacted by the prompt condition and by the gender of the participants. No effects of vocal impressions were found. This section focuses on how the participants evaluated the talker based on the facial information presented and how the participants evaluated the talker influenced their perceptual responses.

**Figure 5:**
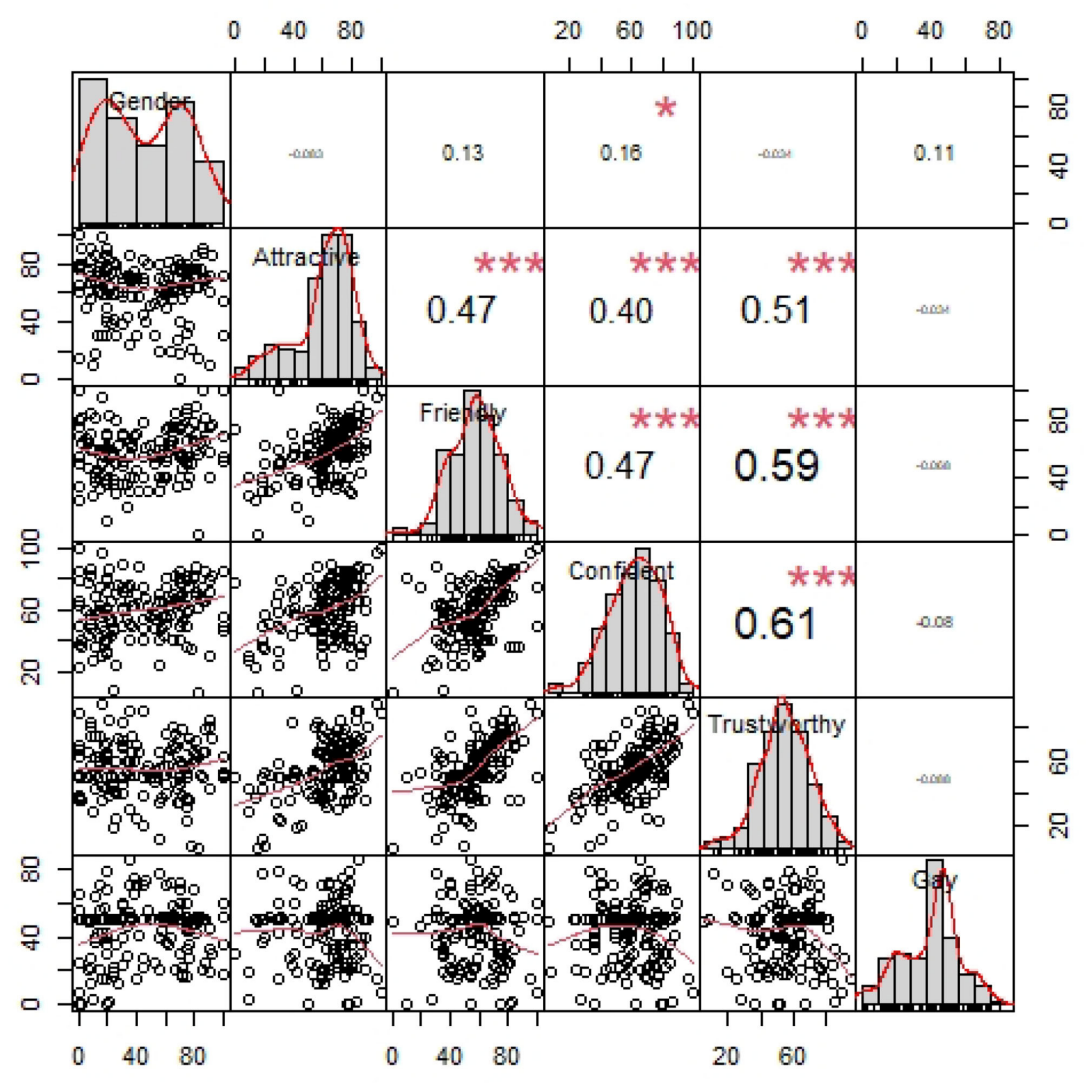
Correlations between the ratings across different visual attributes. Each point corresponds to the ratings of a participant. ^***^p < 0.001; ^*^p < 0.05.

Table 4 summarizes the visual impression ratings. As already noted above, there is quite a bit of variability in gender ratings in both face prompt conditions. This is noteworthy since the face images selected are deemed most gender-prototypical within the Chicago Face Database (Ma et al., 2015). As with the vocal attributes discussed above, there is a great deal of variation in ratings for the other vocal impression dimensions as well as variation in how the attributes relate to each other (see Figure 5). Specifically, among the visual attributes, Attractiveness, Friendliness, Confidence, and Trustworthy are highly positively correlated with each other. There is also a weakly positive correlation between gender prototypicality and confidence. The distributions of the vocal attributes within the visual prompt sub-sample do not differ much from the full sample discussed above. There are strong correlations between Attractiveness, Friendliness, Confidence, and Trustworthy and between Gender and Friendliness.

**Table 4:** Mean ratings (and standard deviations in parentheses) for perceived visual gender, attractiveness, friendliness, confidence, trustworthiness, and gayness in the two face conditions arranged by the gender of the participants.

**Condition**	**Listener**	***N***	**Gender**	**Attractive**	**Friendly**	**Confident**	**Trustworthy**	**Gay**
Female	Female	36	20 (15)	62 (20)	54 (17)	55 (18)	55 (16)	45 (22)
Female	Male	40	20 (13)	64 (20)	56 (18)	61 (20)	57 (16)	36 (19)
Male	Female	35	69 (13)	59 (21)	61 (15)	59 (15)	50 (18)	46 (16)
Male	Male	40	70 (17)	62 (20)	60 (19)	63 (17)	57 (17)	44 (14)

Following the PCA procedure introduced above, we obtained linear combinations of the visual and vocal impression ratings that would capture the maximum variation. The relative weightings and proportion of variance for each component for the visual impression ratings are summarized in Table 5. The optimal linear combination (PC1), which accounts for about 42% of the variance, and the 2nd component (PC2), which accounts for approximately 19% of the variance, were selected as independent variables for the analysis below; the first two components collectively account for around 62% of the variance. PC1 has strong loadings for visual attractiveness, friendliness, confidence, and trustworthiness, which can be interpreted as indexing “visual appeal”. PC2, on the other hand, is dominated by visual gender and gay-looking, which pertain to matters of gender and sexual orientation stereotypes.

**Table 5:** The cumulative proportion of variance accounted for and loadings from the PCA of the visual impression ratings.

	**PC1**	**PC2**	**PC3**	**PC4**	**PC5**	**PC6**
Visual gender	−0.05	0.79	−0.48	0.26	0.16	−0.23
Visual attractiveness	−0.45	−0.15	0.31	0.59	0.57	0.00
Visual friendliness	−0.50	0.10	−0.03	0.32	−0.70	0.38
Visual confidence	−0.49	0.14	−0.15	−0.61	0.36	0.47
Visual trustworthiness	−0.54	−0.08	0.08	−0.30	−0.18	−0.76
Gay-looking	0.08	0.57	0.80	−0.14	−0.07	0.03
Standard deviation	1.60	1.07	0.96	0.75	0.70	0.57
Proportion of variation	0.42	0.19	0.15	0.09	0.08	0.05
Cumulative proportion	0.42	0.62	0.77	0.86	0.95	1.00

Another PCA analysis of the vocal impression ratings was also conducted, focusing on just the vocal impression ratings from participants in the two visual prompt conditions only. The relative weightings and proportion of variance for each component for the vocal attributes are summarized in Table 6. Similar to the PCA of the vocal impression ratings of all three prompt conditions, PC1 has strong loadings for vocal attractiveness, friendliness, confidence, and trustworthiness, while PC2 is dominated by vocal gender, confidence, and gay-sounding.

**Table 6:** The cumulative proportion of variance accounted for and loadings from the PCA of the vocal impression ratings.

	**PC1**	**PC2**	**PC3**	**PC4**	**PC5**	**PC6**
Gender	0.18	0.62	−0.69	0.08	−0.31	0.05
Vocal attractiveness	−0.42	0.05	0.06	0.90	−0.05	−0.02
Vocal friendliness	−0.52	−0.09	0.06	−0.27	−0.60	0.53
Vocal confidence	−0.46	0.40	−0.08	−0.19	0.70	0.31
Vocal trustworthiness	−0.54	0.03	−0.14	−0.27	−0.12	−0.78
Gay-sounding	−0.10	−0.67	−0.70	0.05	0.20	0.12
Standard deviation	1.60	1.07	0.96	0.75	0.70	0.57
Proportion of variation	0.43	0.19	0.15	0.11	0.07	0.05
Cumulative proportion	0.43	0.62	0.77	0.88	0.95	1.00

**Figure 6:**
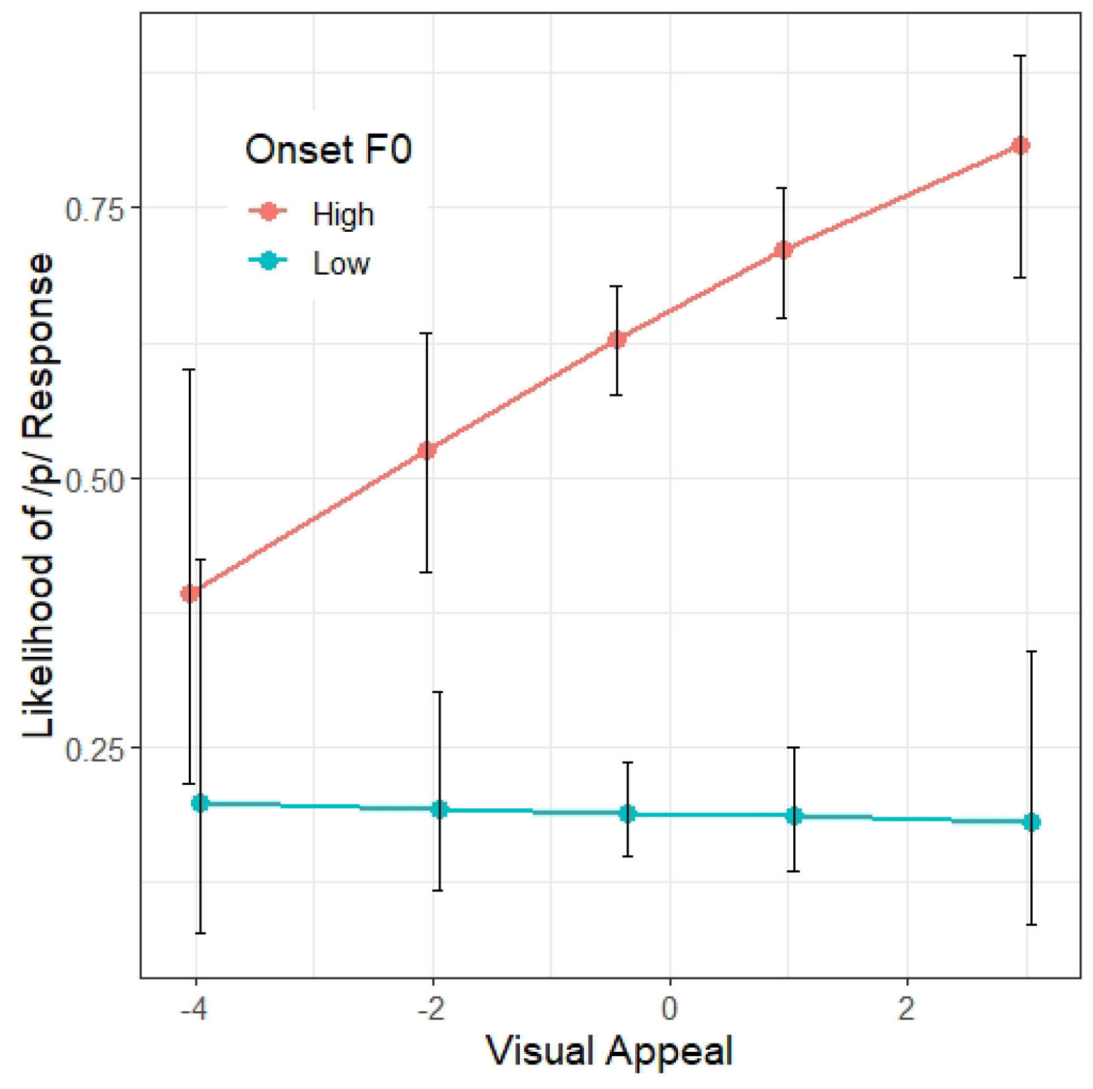
Model 2 predictions of the probability of a /p/ response (y axis) in different F0 onset conditions according to the talker's visual appeal (x axis). The error bars indicate 95% confidence intervals.

**Figure 7:**
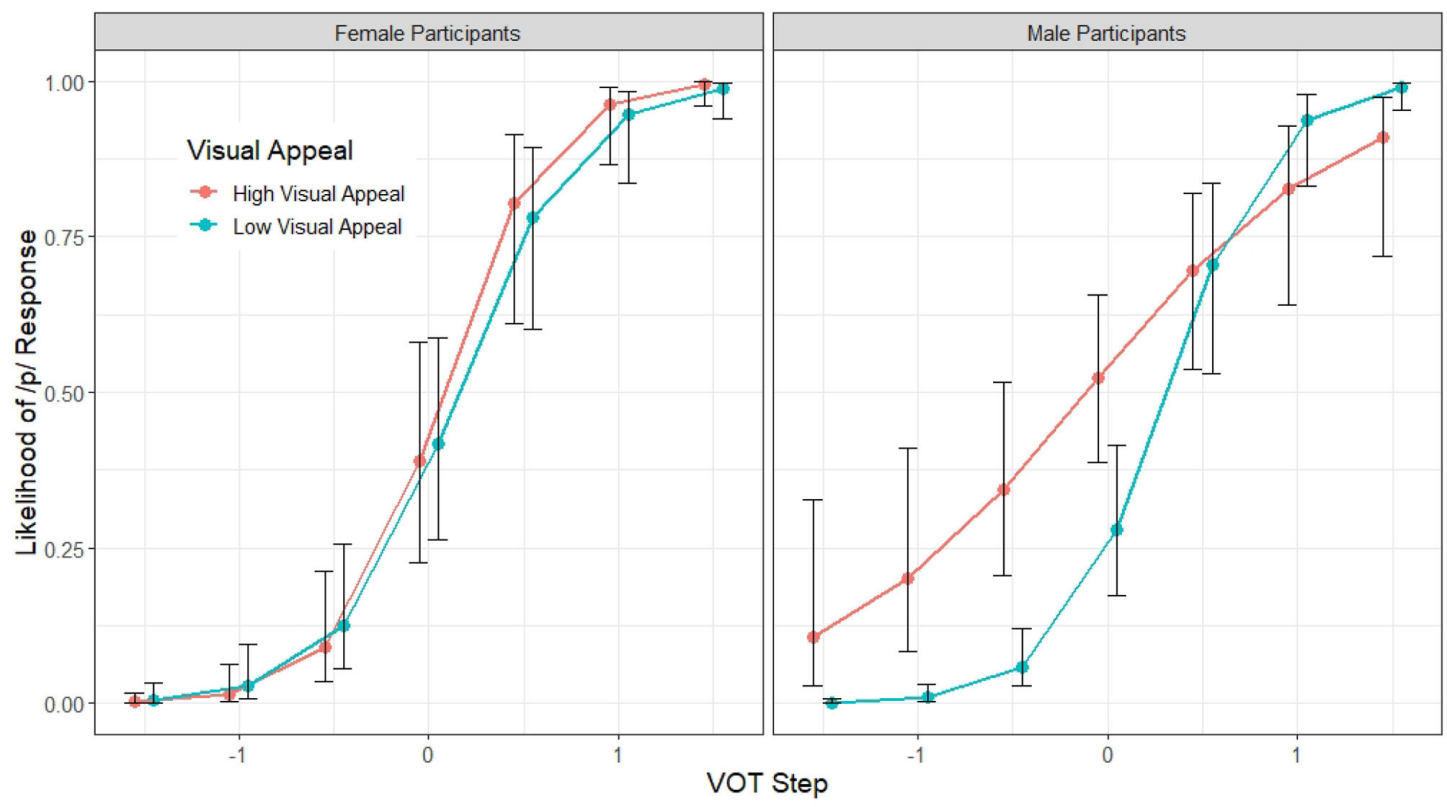
Model 2 predictions of the probability of a /p/ response (y axis) at different steps along the VOT continuum (x axis) according to the participant's gender as well as the talker's visual appeal. While Visual Appeal is continuous, for ease of presentation, only the patterns of talkers with high visual appeal (i.e., 2 standard deviation above the mean) vs. low visual appeal (2 standard deviation below the mean) are shown in the figure. The error bars indicate 95% confidence intervals.

A summary of the second regression model, Model 2, appears in Table 7. The second regression model is similar to the first model in all respects except that the Condition variable was not included; instead, we included the PC1 and PC2 of the visual and vocal impression ratings as discussed above. The signs of the principal components were reversed before entering the model for ease of interpretation (e.g., the higher the PC1 value of the visual impression ratings, the greater the visual appeal). Model selection started with the maximal model with all possible interactions between fixed factors (the impression rating attributes do not interact with each other, however), as well as the random intercepts and slopes, and proceeded by comparing between models with and without the inclusion of an impression attribute and its interaction with other factors. Visual and vocal impression attributes and their interactions that do not improve model likelihood significantly were dropped. In the end, out of the four impression attributes, only PC1 of the visual attributes was retained following this model selection procedure. For ease of reference, PC1 of the visual attributes will be referred to as “Visual Appeal” from hereon. The final model is as follows: Response (pear = 1) ~ Block + F0 * VOT * Gender * Visual Appeal + (1+Block + VOT * F0|Participant). In addition to the main effects of Block, VOT, F0, and the interactions between the latter two, and between VOT and the gender of the participant, Model 2 also revealed several significant Visual Appeal interactions. To begin with, there is a significant interaction between F0 and Visual Appeal (β = 0.15, *z* = 2.45, *p* = 0.01), suggesting that the magnitude of the F0 effect on stop voicing perception is larger for listeners who found the talker visually more appealing (Figure 6). There is a significant interaction between VOT and participant Gender (β = 0.46, *z* = 2.81, *p* < 0.01), but this interaction is mediated by Visual Appeal (β = 0.34, *z* = 2.01, *p* < 0.05). As illustrated in Figure 7, the visual Appeal effect is driven by the behavior of the male participants. Specifically, the more the male participant found the talker visually appealing, the less reliant they are on VOT as a cue for stop voicing perception, as indicated by the shallower VOT slope.

**Table 7:** Estimates for all predictors in Model 2.

	**Model 2**
Intercept	−0.41(0.10)^***^
VOT	3.02(0.16)^***^
F0	1.05(0.06)^***^
Gender	0.02(0.10)
Appeal	0.12(0.11)
Block	0.10(0.04)^**^
VOT:F0	−0.59(0.05)^***^
VOT:Gender	0.46(0.16)^**^
F0:Gender	0.03(0.06)
VOT:Appeal	−0.21(0.17)
F0:Appeal	0.15(0.06)^*^
Gender:Appeal	−0.14(0.11)
VOT:F0:Gender	−0.10(0.05)
VOT:F0:Appeal	−0.03(0.05)
VOT:Gender:Appeal	0.34(0.17)^*^
F0:Gender:Appeal	0.08(0.06)
VOT:F0:Gender:Appeal	−0.10(0.05)^*^
AIC	12098.96
BIC	12346.50
Log Likelihood	−6017.48
Num. obs.	16912
Num. groups: Participant	151
Var: Participant Intercept	1.47
Var: Participant Block	0.10
Var: Participant F0	0.39
Var: Participant VOT	3.45
Var: Participant F0:VOT	0.13

*Gender refers to the gender of the participant; Appeal refers to the PC1 of the visual impression ratings*.

*** *p < 0.001*;

** *p < 0.01*;

* *p < 0.05*.

**Figure 8:**
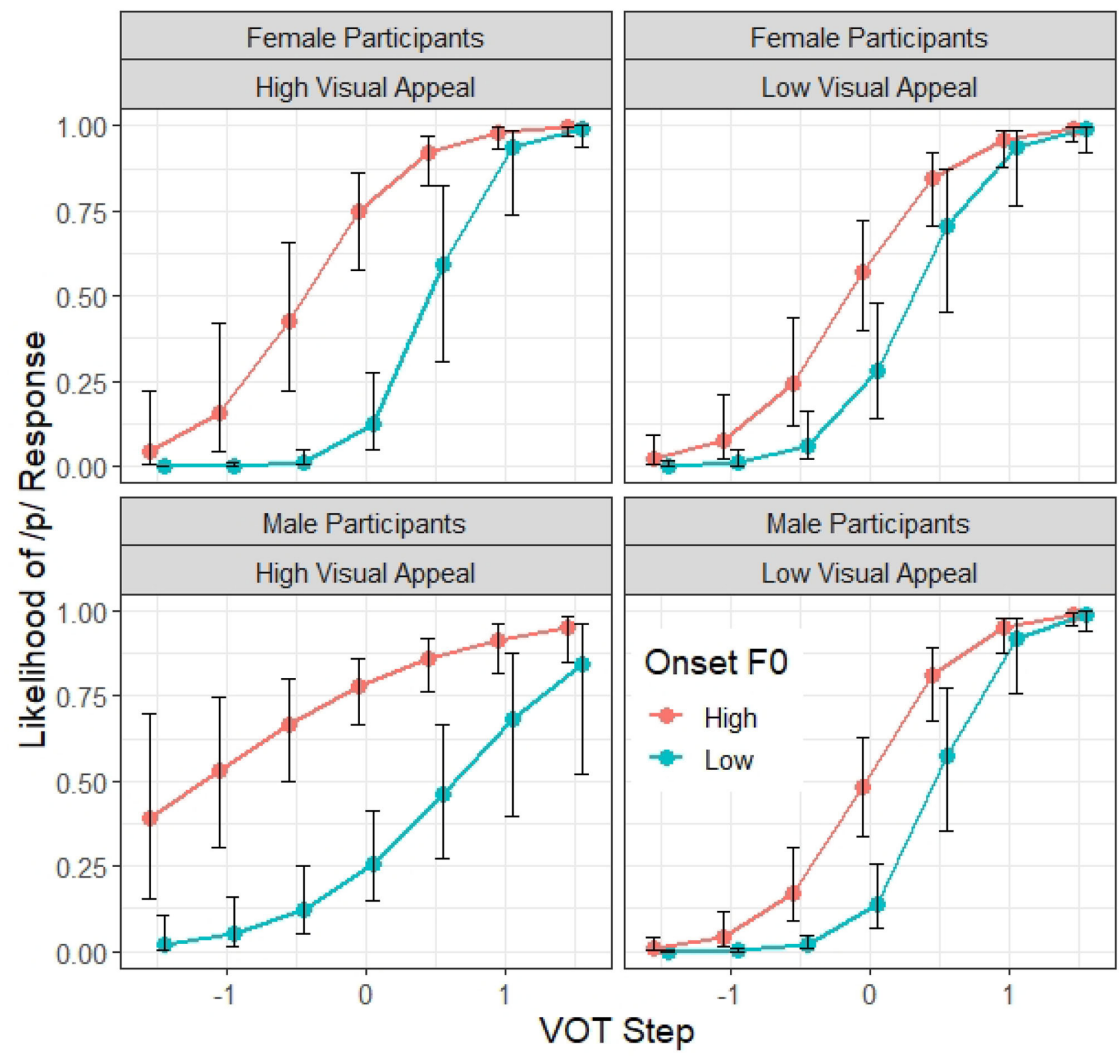
Model 2 predictions of the probability of a /p/ response (y axis) at different steps on the VOT continuum (x axis) and F0 targets according to the talker's visual appeal and the gender of the participant. While Visual Appeal is continuous, for ease of presentation, only the patterns of talkers who were rated by the participated as having high visual appeal (i.e., 2 standard deviation above the mean) vs. those with low visual appeal (2 standard deviation below the mean) are shown in the figure. The error bars indicate 95% confidence intervals.

Finally, there is also a significant four-way interaction between VOT, F0, participant Gender and Visual Appeal (β = −0.10, *z* = −2.11, *p* < 0.05). As illustrated in Figure 8, male and female listeners who rated the talker as having lower visual appeal do not differ very much in terms of their patterns of /p/ response across the VOT and F0 conditions. However, for the participants who rated the talker as having greater visual appeal, they are more likely to rely on the F0 cue (as indicated by the larger difference in /p/ response between the two onset F0 conditions) and less reliant on VOT information (as indicated by the shallower slope of the identification function along the VOT dimension). However, this visual appeal difference is more robust among the male listeners than the female listeners.

## 5. Discussion

This study examined the effects of a listener's gender and his/her perception of a talker's gender and paralinguistic attributes on perceptual cue weighting using a matched-guise paradigm. Gender-neutral stimuli were presented to three groups of listeners, one group saw a prototypical male face, one saw a prototypical female face, and one without any visual prompt. Our regression analyses revealed that listeners who saw a male face showed less reliance on VOT compared to the listeners who saw a female face or were given no visual information. Male listeners are also less reliant on VOT in stop voicing classification. Listeners' visual impression of the talker also affected their weighting of the VOT and F0 cues. When visual information is available, listeners who had a favorable impression of the talker were less likely to rely on VOT and more likely to pay attention to the F0 cue in stop voicing classification. Male listeners who rated the talker as having more visual appeal showed the stronger reliance on F0 and the least reliance on the VOT cue.

While our findings show that perceptual cue weighting is influenced by the listener's gender and the subjective evaluations of the speaker by the listener, the mapping between the participant's interpretation of the talker's paralinguistic attributes does not always map onto the participants' perceptual responses to the VOT and onset F0 cues in the predicted manner. As noted above, to the extent that attractiveness and confidence are associated with greater VOT contrast realization, we had anticipated that listeners who rated the talker as more attractive and confident would rely more on the VOT cue than the onset F0 cue. Likewise, to the extent that femininity, friendliness, trustworthiness, and gayness are associated high mean F0 and more exaggerated F0 excursions, we had expected that listeners who rated the talker higher along these dimensions to rely more on the onset F0 cue than the VOT cue since onset F0 has been found to be more exaggerated when global F0 is high. Our results suggest that the influence of the participants' subjective impressions of the talker on VOT/F0 cue reliance is much more nuanced. To begin with, there are strong positive correlations between impressionistic judgments that were predicted to have opposite effects on cue weighting. That is, attractiveness, confidence, friendliness and trustworthiness are highly correlated even though the first two attributes were predicted to be positively associated with greater VOT reliance while the latter two attributes are associated with weaker VOT reliance. The cue-combination analytic approach adopted in the analysis (i.e., the use of Principal Component Analysis to reduce the number of highly correlated parameters prior to further modeling) prevents a direct mapping between impressionistic ratings and the participants' perceptual responses. In the end, we found that the participants would rely more heavily on the onset F0 cue than the VOT cue when the talker is rated as having greater visual appeal, a principal component involving strong loadings of attractiveness, friendliness, confidence, and trustworthiness. This state of affair points to the complexity in the way impressionistic judgments formed by the listeners interacted with the listeners' speech perceptual processes. While the Frequency Code and Effort Code hypotheses suggest potential universal associations between paralinguistic information and speech cues, it is unlikely that all associations between subjective evaluations and speech cues are fully translatable across individuals, speech communities, and cultures. Babel and McGuire (2013), for example, found that, even though perceived attractiveness ratings are highly correlated across three different varieties of North American English, listener populations nonetheless differed in the phonetic features used to make attractiveness judgments, suggesting that vocal attractiveness is dependent on community-specific preferences. Our findings suggest that more nuanced research is needed to elucidate the complex interplay between a listener's subject evaluation of his/her interlocutor and the way the listener perceives the speech outputs of that interlocutor.

Our findings are consistent with the idea that first impressions of a person can have subtle and often subjectively unrecognized effects on subsequent deliberate judgments, including perceptual cue weighting in a stop voicing classification task. The fact that visual appeal, rather than vocal appeal, exerts a stronger influence on perceptual cue weighting, as evidenced by the results of Model 2, is surprising *a priori* given the close connection between the speech cues and vocal impressions. Our findings suggest that listeners might, in general, rely more on visual impression than vocal ones to inform their perceptual judgments. Indeed, other studies have also reported stronger effects of facial impressions over vocal ones (e.g., Hou and Ye, 2019). In one study, the influence of visual impression is nearly triple that of vocal impression when evaluating competence (Klofstad, 2017). Recent models of social cognition and decision-making (Chaiken and Trope, 1999; Kahneman, 2003) posit a dual process where fast, unreflective, effortless “system 1” processes contrasts with slow, deliberate, effortful “system 2” processes. Inferences from facial appearance have been characterized as system 1 processes (Winston et al., 2002; Todorov et al., 2005). To be sure, the stronger effect of visual impression might also have stemmed from the particular design of this study. Participants in the visual prompt conditions were asked to evaluate the talker visually first prior to the talker's vocal information being introduced. Thus, the participant's earliest first impressions of the talker were formed entirely based on visual information alone. First impressions based on visual cues alone might have a stronger biasing effect on the subsequent behavior of the listeners than the vocal information which was introduced later. The gender-specificity of the effects of visual impressions on cue weighting is also noteworthy. The effects of visual appeal, as revealed in Model 2, is more strongly driven by the male participants. These findings are consistent with the observation that men and women may be affected by their own impressionistic judgments differently. For example, men evaluate female facial attractiveness as higher than male facial attractiveness while women do not show a similar tendency in evaluation male facial attractiveness higher than female facial attractiveness (Hou and Ye, 2019). Babel (2012) found that men and women exhibit different rates of vowel imitation depending on the race and attractiveness of the talker.

The fact that a listener's perception of the gender and personality features of a talker could affect the listener's cue weight raises question about the mechanism(s) behind such an influence. As noted earlier, exemplar-based models of speech perception and production that allow socio-indexical information to be encoded as part of the episodic traces in the mental lexicon provides a potential model for understanding how socio-indexical and paralinguistic information could modulate speech perception. We hypothesized that, when a listener judges a talker to be of a particular gender or has certain personality features, the listener's perceptual system might adjust its cue weight expectation in accordance to the specific socio-indexical and paralinguistic norms. Our findings are broadly consistent with these predictions. Specifically, the direction of the cue weight adjustments with respect to perceived gender is consistent with the idea that listeners are informed by the past experiences (i.e., VOT distinctions among oral stops are less distinct among males than among females). Male participants exhibited the strongest cue weight adjustments, presumably due to their familiarity with their own production tendencies relative to their female counterparts. As noted above, the influence of impressionistic judgments on the personality attributes of the talker on cue weights are more nuanced due to the complex mapping between VOT and F0 variations and personality traits. The final analysis suggests that when the participant found the talker to have high visual appeal, the participant is more likely to pay greater attention to onset F0 than VOT cues. This pattern is consistent with the observation that higher pitch is associated with more affective interpretations. That is, if individuals with greater visual appeal are seen as more affective people, great visual appeal might have primed the participants to activate perceptual experiences associated with affective individuals. Listeners might heighten their attention to onset F0 differences since affective individuals are associated with higher overall F0 and less distinct VOT contrast in their speech outputs.

The fact that native English-speaking listeners' perceptual weighting of VOT and onset F0 cues is impacted by the perceived socio-indexical and personality characteristics of the talker lends further support to the idea that the relationship between the VOT and onset F0 cues in English is part of the controlled phonetic knowledge of English speakers (Kingston and Diehl, 1994; Solé, 2007). According to the cue-reweighting approach to the development of tone split and tonogenesis (Hyman, 1976; Kang, 2014; Coetzee et al., 2018), one pathway to developing allophonic pitch variation is via the phonologization of consonantal perturbation of pitch on the neighboring vowel. The fact that the trading relation between VOT and onset F0 is part of the phonetic knowledge of English speakers raises the question of whether English might be undergoing a sound change in progress. That is, are English stops developing a tone split analogous to what has been documented in Afrikaans (Coetzee et al., 2018) recently? While this is not a question the present study can answer definitively, it is nonetheless important to note that, given the propagation of any sound change crucially depends on the innovative variation developing sociolinguistic significance, the fact that English-speaking listeners are sensitive to the social characteristics of the talker in their perceptual responses to VOT/F0 variation points to, at the minimum, the emergence of some form of sociolinguistic awareness of the VOT/F0 covariation. This interpretation is further supported by developmental studies that look at gender differentiation in VOT realization. Whiteside and Marshall (2001), for example, studied the developmental trajectory of VOT in English /p/ and /t/ for boys and girls aged 7, 9, and 11 years and found that mean VOT differences between voiced and voiceless stops were larger for girls than for boys at age 11 due to the boys' marked decrease in VOT difference from age 9 to 11. They argued that the gender differences might be the result of the amplification of an intrinsic variation due to anatomical differences between males and females. To be sure, it is not clear at this point if comparable onset F0 changes would accompany the gender-differentiated developmental changes in VOT, but our findings suggest that, at least among adult listeners, the trading relationship between VOT and onset F0 is gender-differentiated. These gender differences in the production and perception of VOT/onset F0 variation are prime materials (i.e., the first order indexicality association) for the speakers to recruit in their ideological projects (Eckert, 2019). What is observed in English today might be an analog to the precursor stage to the development of F0 distinctions in the Seoul Korean stop laryngeal system. Oh (2011), for example, examined the VOT of voiceless aspirated plosives in Seoul Korean and found that male speakers have significantly longer VOT than female speakers. She hypothesized a potential link between the gender difference to an ongoing change where the distinction between lenis and aspirated plosives are increasingly cued by differences in F0 rather than VOT. While no definitive historical evidence was provided, she did note that the gender difference appeared to have existed prior to the sound change commencing in Seoul Korean (see also Kang, 2014).

In sum, the present study offers crucial evidence for listeners' sensitivity to the talker's socio-indexical and personality characteristics in their perceptual responses to VOT and onset F0 variation. Our findings lend support for the type of cue reweighting model of sound change (Hyman, 1976; Kang, 2014; Coetzee et al., 2018), as they not only further cement the controlled phonetic knowledge interpration of VOT/onset F0 co-variation in English, but also reveal a sociolinguistic dimension to this co-variation. More investigation is needed to examine the possibility of a sound change in progress in North American English concerning the relation between stop voicing and F0. In particular, apparent time investigations or panel studies into the community patterning of the F0 perturbation effect in North American English across age groups and gender could be particularly revealing.

## Data Availability Statement

The datasets presented in this study can be found in online repositories. The supplementary materials only provide the images and sound files used. The dataset and the analysis scripts can be found at https://osf.io/fx8ay/.

## Ethics Statement

The studies involving human participants were reviewed and approved by The Social and Behavioral Sciences Institutional Review Board at the University of Chicago. The patients/participants provided their written informed consent to participate in this study.

## Author Contributions

AY contributed to the design and implementation of the research topic, to the analysis of the results, and writing of the manuscript.

## Funding

This work was supported in part by the National Science Foundation (BCS1827409).

## Conflict of Interest

The author declares that the research was conducted in the absence of any commercial or financial relationships that could be construed as a potential conflict of interest.

## Publisher's Note

All claims expressed in this article are solely those of the authors and do not necessarily represent those of their affiliated organizations, or those of the publisher, the editors and the reviewers. Any product that may be evaluated in this article, or claim that may be made by its manufacturer, is not guaranteed or endorsed by the publisher.
