# Supplementary material for: Perceptual Cue Weighting Is Influenced by the Listener's Gender and Subjective Evaluations of the Speaker: The Case of English Stop Voicing
Source: Front Psychol. 2022 Apr 20;13:840291. doi: 10.3389/fpsyg.2022.840291 (PMC9067435; doi:10.3389/fpsyg.2022.840291)
Supplement: Supplementary file 2 [file Data_Sheet_2.docx]

Reproduced with permission from Chicago Face Database, available at https://www.chicagofaces.org/download/.


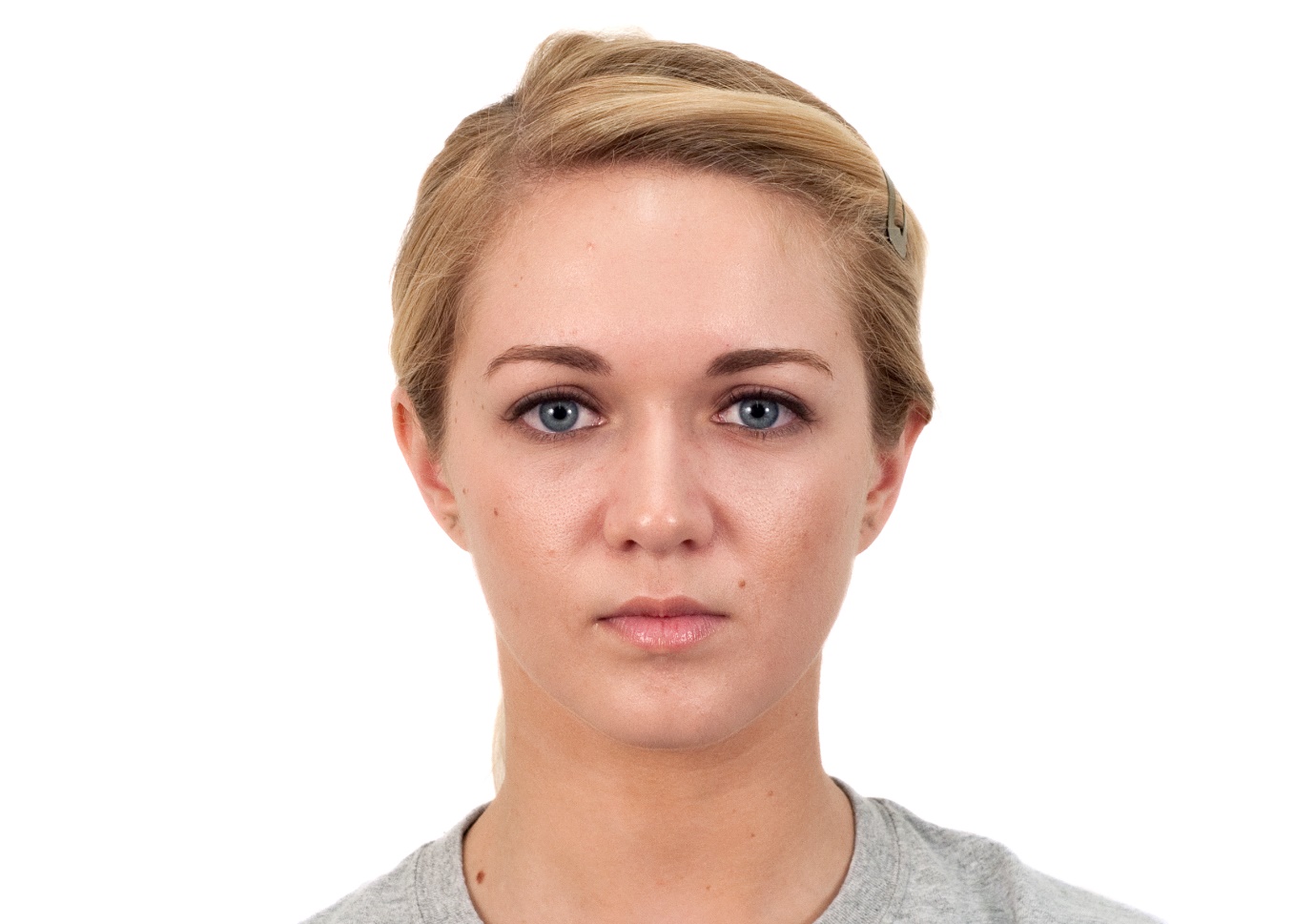


Figure S1: CFD-WF-233-112-N.jpg; image from the Chicago Face Database used in the Female Talker Prompt Condition


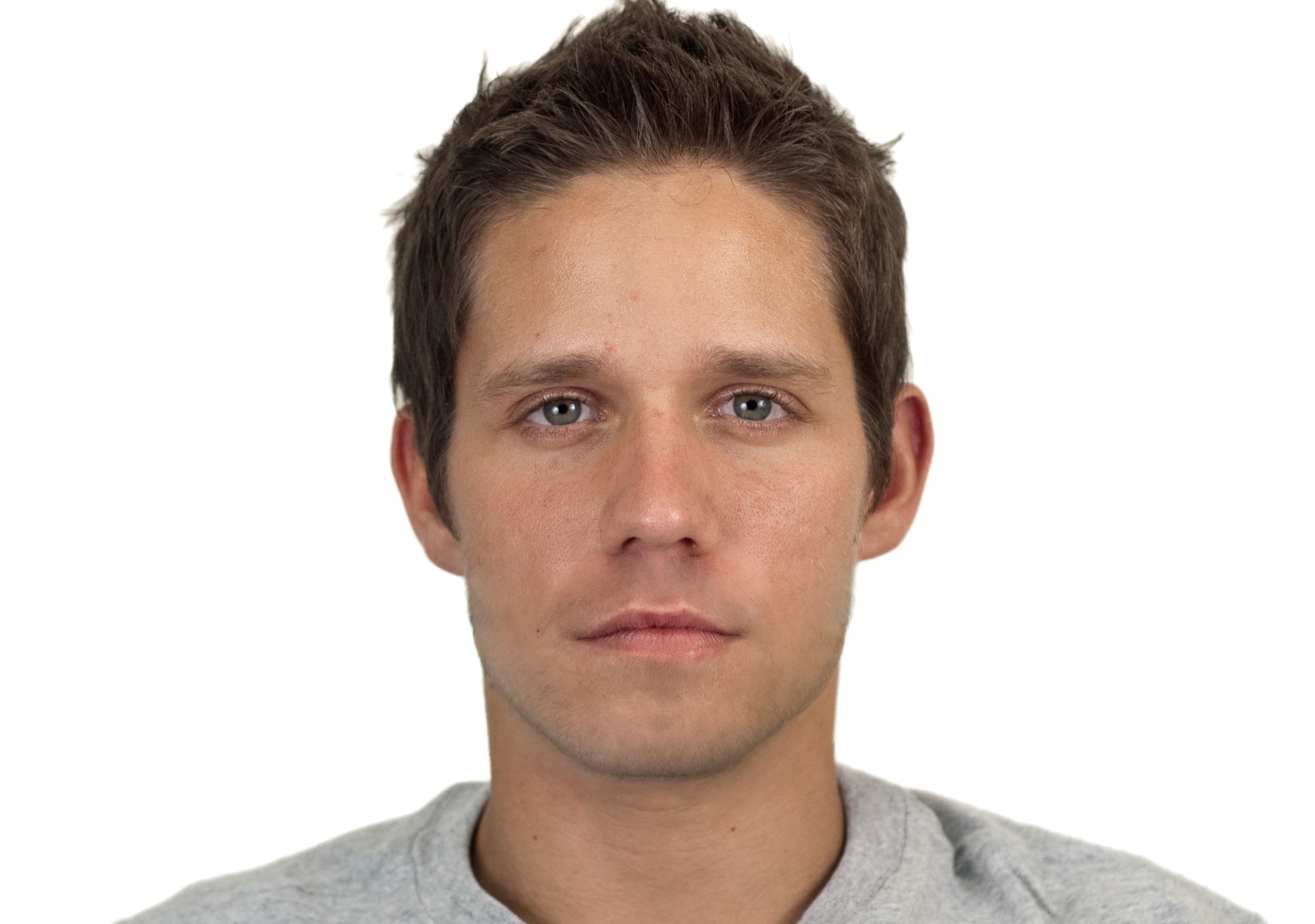


Figure S2: CFD-WM-004-010-N.jpg; image from the Chicago Face Database used in the Female Talker Prompt Condition
